# Metabolic Dynamics of Developing Rice Seeds Under High Night-Time Temperature Stress

**DOI:** 10.3389/fpls.2019.01443

**Published:** 2019-11-08

**Authors:** Balpreet K. Dhatt, Nathan Abshire, Puneet Paul, Kalani Hasanthika, Jaspreet Sandhu, Qi Zhang, Toshihiro Obata, Harkamal Walia

**Affiliations:** ^1^Department of Agronomy and Horticulture, University of Nebraska-Lincoln, Lincoln, NE, United States; ^2^Department of Biochemistry, University of Nebraska-Lincoln, Lincoln, NE, United States; ^3^Department of Statistics, University of Nebraska-Lincoln, Lincoln, NE, United States

**Keywords:** rice, early seed development, grain filling, high night-time temperature, metabolite profiling, starch

## Abstract

High temperature stress during rice reproductive development results in yield losses. Reduced grain yield and grain quality has been associated with high temperature stress, and specifically with high night-time temperatures (HNT). Characterizing the impact of HNT on the phenotypic and metabolic status of developing rice seeds can provide insights into the mechanisms involved in yield and quality decline. Here, we examined the impact of warmer nights on the morphology and metabolome during early seed development in six diverse rice accessions. Seed size was sensitive to HNT in four of the six genotypes, while seed fertility and seed weight were unaffected. We observed genotypic differences for negative impact of HNT on grain quality. This was evident from the chalky grain appearance due to impaired packaging of starch granules. Metabolite profiles during early seed development (3 and 4 days after fertilization; DAF) were distinct from the early grain filling stages (7 and 10 DAF) under optimal conditions. We observed that accumulation of sugars (sucrose, fructose, and glucose) peaked at 7 DAF suggesting a major flux of carbon into glycolysis, tricarboxylic acid cycle, and starch biosynthesis during grain filling. Next, we determined hyper (HNT > control) and hypo (HNT < control) abundant metabolites and found 19 of the 57 metabolites to differ significantly between HNT and control treatments. The most prominent changes were exhibited by differential abundance of sugar and sugar alcohols under HNT, which could be linked to a protective mechanism against the HNT damage. Overall, our results indicate that combining metabolic profiles of developing grains with yield and quality parameters under high night temperature stress could provide insight for exploration of natural variation for HNT tolerance in the rice germplasm.

## Introduction

Rice (*Oryza sativa*) is a staple food crop for over half the world’s population, especially in Asia where 90% of rice is produced and consumed and provides 50% of the dietary caloric supply (Khush, 2005; Khush and Jena, 2009; Muthayya et al., 2014). Rise in rice production, attributed to the green revolution, has slowed in recent years (Ray et al., 2012; Grassini et al., 2013). Plateauing of rice yields has been reported for major rice producing countries including China, India, and Indonesia (Ray et al., 2012). Furthermore, diminishing arable land, less water, and increased frequency of extreme weather events are adding to the food security challenges (Foley et al., 2011; Röth et al., 2016).

Increase in temperature is one of the most detrimental factor affecting yields of major crops such as rice, maize, and wheat (Zhao et al., 2017). High temperature stress (HTS), especially during reproductive development, results in high level of spikelet sterility due to failure of either pollen development, pollen tube elongation or fertilization (Prasad et al., 2006; Jagadish et al., 2010; Zinn et al., 2010; Bokszczanin and Fragkostefanakis, 2013; Ali et al., 2019). After fertilization, endosperm undergoes free nuclear divisions (syncytial phase) followed by cell wall formation (cellularization) (Brown et al., 1996). HTS during this developmental period is reported to alter mature seed size (Folsom et al., 2014; Chen et al., 2016; Begcy et al., 2018). Afterwards, developing seed enters grain filling phase where the incoming photo assimilates are used for synthesis of storage compounds such as starch, proteins, and lipids (Xing and Zhang, 2010; Zuo and Li, 2014). Biosynthesis of these compounds is heavily dependent on various metabolic processes and predominantly defines mature seed weight (Tashiro and Wardlaw, 1991a; Tashiro and Wardlaw, 1991b; Schnyder, 1993; Hu et al., 2016; Abayawickrama et al., 2017). HTS during grain filling not only impacts the final grain weight but also leads to structural abnormalities in starch granules, which causes an overall increase in chalkiness, thus lowering the rice quality for human consumption (Lisle et al., 2000; Fitzgerald and Resurreccion, 2009; Ishimaru et al., 2009; Tsutsui et al., 2013). These abnormalities have been linked to insufficient supply of nutrients to the developing endosperm, reduction in starch synthesis or premature degradation of starch (Sato and Inaba, 1976; Yamakawa et al., 2007; Ishimaru et al., 2009).

Further, in context of increasing temperatures, it has been reported that daily minimum temperatures are increasing at a faster rate than daily maximum temperatures in many rice growing regions of Asia (Peng et al., 2004). Not only higher day-time temperatures but HNT are also correlated with decline in rice yield and quality (Morita et al., 2005; Cheng et al., 2009; Ishimaru et al., 2009; Mohammed and Tarpley, 2011; Shi et al., 2013; Coast et al., 2015) Night-time temperatures as low as 29°C (33/29°C; day/night) caused significant reduction in rice yield (Ziska and Manalo, 1996; Shi et al., 2013; Bahuguna et al., 2017). This decline in grain yield was attributed to increased respiratory metabolism during night-time leading to carbon loss (Bahuguna et al., 2017). Moreover, HNT during grain filling phase in rice hinders translocation and accumulation of photo-assimilates, thereby altering grain weight, width, and quality (Shi et al., 2013). A recent study on the impact of HNT on winter wheat indicated that a tolerant wheat cultivar accumulated major sugars exclusively in the spikes during early stages of seed development in contrast to the sensitive cultivars (Impa et al., 2019). This study also reported accumulation of the sugar, trehalose, under HNT in both sensitive and tolerant winter wheat. Since, trehalose is closely related with trehalose-6-phosphate, which is involved in sugar signaling, this work suggested the involvement of sugar signaling in the HNT response during early seed development (Impa et al., 2019). Rice response to HNT at metabolic levels have been reported for leaves, where accumulation of amino acids, organic acids, sugars, polyols, and putrescine, as well as mis-regulation of the TCA cycle was observed (Glaubitz et al., 2015; Glaubitz et al., 2017). Although, these results indicate the influence of sugar metabolism on the rice yield under HNT, the effect of HNT on metabolite profiles of developing seeds remain less explored.

In the current study, we investigated the impact of HNT during seed development on morphology and metabolite profile of six rice accessions. Our results indicated alteration in mature seed size and structural impairment of starch granules due to HNT, whereas no differences were observed with respect to percentage fertility and seed weight. The time-course metabolite profiling indicated a differential shift in seed metabolic profiles from the early seed developmental stages coinciding with transition of endosperm from syncytium to cellularization (3 and 4 DAF), as well as early grain filling stages (7 and 10 DAF). We also observed the effect of HNT on sugar metabolism despite the minor effect of HNT on final grain yield parameters. The results with respect to the impact of HNT on physiology and metabolome of rice seed are discussed.

## Materials and Method

### Plant Material and Growth Conditions

We selected six rice accessions from a rice diversity panel (RDP1) based on the available agronomic and geographical information about accessions in the panel (**Table 1**). The selection was based on similar flowering time, with priority for flowering within 120 days so that different accessions were not flowering under divergent light conditions in the greenhouse (Zhao et al., 2011). Seeds from six accessions were germinated in dark on half-strength MS media. The germinated seedlings were transplanted to soil in pots (4 inch square) and the plants were grown in controlled greenhouse conditions, 16 h light and 8 h dark at 28 ± 1 °C and 23 ± 1 °C, respectively, and a relative humidity of 55–60%. During flowering, florets were marked at the time of fertilization to track the precise developmental progression as described in Folsom et al. (2014). One day after fertilization (DAF), plants were either moved to an adjacent greenhouse set for high night temperature treatment (HNT; 16 h light and 8 h dark at 28 ± 1 °C and 28 ± 1 °C; **Figure S1**) or kept under control conditions (as mentioned above). The plants were either used for sampling at specific time points based on days after fertilization (as marked) or allowed to mature in the control and HNT greenhouse for final grain yield and quality data collection.

**Table 1:** Genetic and geographical information of the rice genotypes.

GSOR Id	NSFTV Id	IRGC Id	Name	Sub-population	Country of origin
301163	172	117944	Zhenshan 2	*indica*	China
301110	118	117828	Oro	*temp. jap.*	Chile
301195	204	117860	Razza 77	*temp. jap.*	Italy
301217	226	117762	IRAT 44	*trop. jap.*	Burkina Faso
301369	386	117839	Palmyra	*trop. jap.*	United States
301374	391	117706	Della	*trop. jap.*	United States

Genetic Stocks Oryza collection identification number’ (GSOR Id); National Science Foundation ‘Exploring the genetic basis of transgressive variation in rice’ accessions identification number (NSFTV Id); International Rice Genebank Collection identification number (IRGC); temp. jap. – temperate japonica; trop. jap. – tropical japonica.

### Analysis of Morphometric and Agronomy Traits

To precisely assess the impact of HNT, only florets marked at the time of fertilization (anthesis) were evaluated for downstream analysis: i) fresh/dry weight of developing seeds and ii) percentage of fully developed seeds, weight per seed and morphometric measurements of mature seeds. For analyzing fresh and dry weight, marked developing seed (with husk) at 4, 7, and 10 DAF were collected from control and HNT treated plants. Fresh weights were measured immediately after collection, and dry weights were measured after drying the seeds for 7 days at 40 ˚C for uniform drying. For fresh and dry weight measurements, the sum of 10 developing seeds derived from three plants for each respective time-point and treatment was considered. For analyzing mature seed parameters, plants were grown under either control or HNT conditions until maturity. The mature panicles were harvested and dried for 7 days at 28 °C. For calculating the percentage of fully developed mature seeds, fully filled (as fertile) and unfilled seeds (as sterile) were scored. Only florets marked at the time of fertilization were analyzed at maturity (*n* = 300-600 from 8 to 10 plants per treatment). Morphometric measurements were performed on the marked mature seeds after dehusking; seeds were scanned using Epson Expression 12000 XL scanner (600 dpi resolution) and analyzed by SmartGrain (Tanabata et al., 2012).

### RNA Extraction and RT-qPCRs

SuperScript VILO cDNA synthesis kit (Invitrogen) was used for cDNA synthesis using one µg of total RNA (Qiagen’s RNeasy Plant Mini kit) treated with DNase I (Qiagen). SYBR Green Master Mix (Bio-Rad) was used for RT-qPCR reactions using the Lightcycler 480 Real-Time PCR System (Roche). Ubiquitin (*UBQ5*) gene were used as reference gene (Jain et al., 2006). Data were analyzed using standard methods (Livak and Schmittgen, 2001). For all RT-qPCR assays, three independent biological replicates and three technical replicates were included. Primers used in the study are listed in **Table S5**.

### Scanning Electron Microscopy

For scanning electron microscopy (SEM), rice grains were transversely cut, then placed on conductive tape attached to aluminum SEM stubs and vacuum dried in a sample drying oven at 40 °C for 1 week. Three independent biological replicates (mature seeds) were considered for the observations. Dried samples were sputter coated with chromium using a Denton Vacuum Desk V sputter coater and imaged with Hitachi-S4700 Field-Emission SEM at 50X, 200X, and 2000X. Representative images from only 2000X are shown.

### Metabolite Profiling

The marked developing seeds (with husk) corresponding to 3, 4, 7, and 10 DAF from five biological replicates were harvested from the six rice accessions subjected to control and HNT treatment (**Figure S1**). The harvested tissue was immediately frozen in liquid nitrogen and then transferred to -80 °C. Frozen samples were ground with pestle and mortar. Aliquots of 25 mg powder were used for metabolite extraction by the methanol/water/chloroform method described by Lisec et al. (2006). Two hundred microliters of the polar (upper) phase were dried by vacuum concentrator and derivatized by methoxyamine hydrochloride (Sigma-Aldrich, Milwaukee, WI) and N‐Methyl‐N‐(trimethylsilyl) trifluoroacetamide (CovaChem, Loves Park, IL) as described by Lisec et al. (2006). A reference sample was made by combining 5 µl aliquots from all samples. Gas chromatography was performed using an 7200 GCQTOF System (Agilent Technologies, Santa Clara, CA, United States) with HP-5MS UI GC Column (30 m × 0.25 mm, 0.25 µm; Agilent), helium carrier gas (Ultra High Purity 99.999%, Matheson, Lincoln, NE) at a flow rate of 1 ml min^-1^, an injection volume of 1 µl by split 10 and 100 settings, and the GC oven temperature program consisting of initial temperature 80°C with a hold time of 2 min, with a ramp of 15°C min^-1^ and a final temperature of 330°C with a hold time of 6 min. Electron ionization was performed with an EI energy of 70 ev and an ion source temperature of 280°C. Mass spectrometry was performed in TOF mode with the transfer line temperature set to 280°C, the range of acquired mass spectra from 70 to 600 amu, and a 50 Hz scan rate. Peaks from the reference sample were annotated by the Agilent Masshunter Unknown Analysis software using the Fiehn GC/MS Metabolomics RTL Library (G1676AA; Agilent) to produce a batch specific metabolite library file for peak annotation and peak area quantification in all individual samples by the Masshunter QTOF Quantitative Analysis software (Agilent). See **Table S4** for ion m/z and retention indexes used for metabolite annotation and quantification. After blank subtraction, peak area of representative ion was normalized by that of ribitol added as an internal standard and by the fresh weight of the sample to represent relative levels of metabolites.

### Statistical Analysis

To examine the temporal effect on metabolite levels, we applied R/Bioconductor package *limma* only to the control dataset using time as a categorical variable (Ritchie et al., 2015). We evaluated the difference between each pair of adjacent time points (3–4, 4–7, and 7–10 DAF) and their statistical significance. Using a cutoff in adjusted p-value at 0.05, we labeled the significantly different changes as either “up” or “down” regulated based on the direction of their change, with all the statistically insignificant changes labeled as “stable”. Then, we grouped metabolites based on their label sequence (three labels for each metabolite) so that metabolites in the same group had similar trends.

Next, we determined the effect of HNT on the metabolic profile during early seed development (3 and 4 DAF) and early grain filling stages (7 and 10 DAF). For this, we normalized all metabolite levels by taking the ratio for each metabolite (averaged across genotypes) in control group for the first time point (3 DAF) as denominator and applied a log transformation. To detect the HNT effect, we used *limma*, which implements a linear model originally designed for differential expression analysis of microarray (Ritchie et al., 2015). Additionally, we included time as a categorical covariate as there were only four time points (3, 4, 7, and 10 DAF). We fitted the *limma* model separately for each genotype. To combine these models, we also conducted a meta-analysis using R package *metap* (Dewey, 2019). *metap* takes the p-values from the previous analysis and the directions of the effects; whether it was hyper (HNT > control) or hypo abundant (HNT < control), as input. The output from this analysis is a single p-value for the overall effect. For the metabolites that were hyper- or hypo- abundant, we performed a one-sided test enforcing the direction of effect with the sign of log fold change values, but for the others we used a two-sided test. We removed two metabolites prior to the meta-analysis, D-mannitol, and ecosapentaenoic acid, as their super-high abundance at or around 10 DAF affected the analysis.

From both these approaches, we were able to i) derive clusters of metabolites with significant temporal variation pattern during the seed developmental stages, and ii) obtain a conservative list of metabolites with statistically significant HNT effect. To put these results in the broader context, we also performed hierarchical clustering analysis (HCA) on the temporal dataset as well as the HNT/control log ratio data using R package pheatmap (Kolde, 2015).

## Results

### Impacts of HNT on Agronomic Traits and Grain Morphology

**Figure 1:**
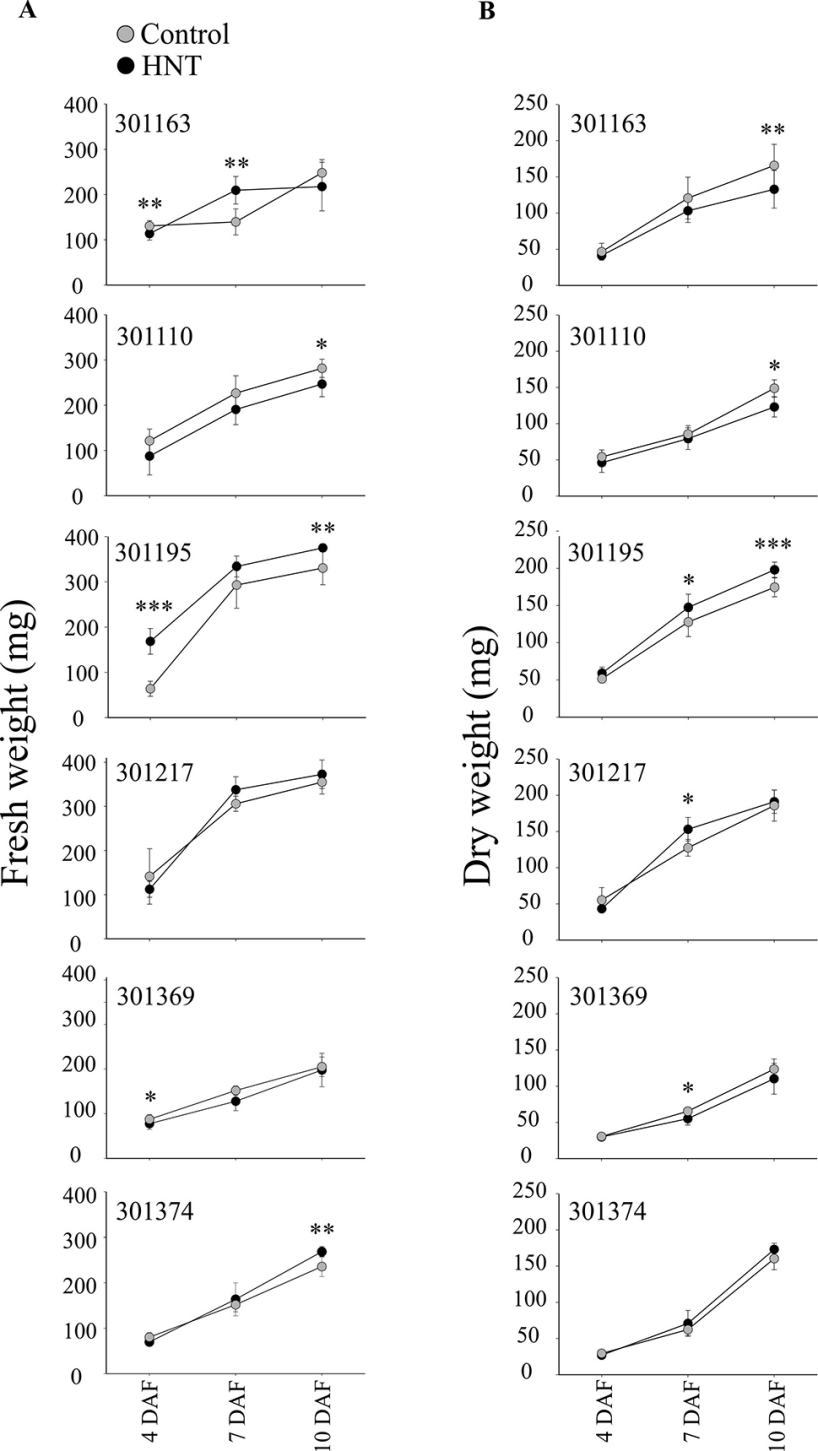
Fresh and dry weight analysis of developing seeds. Florets were marked at the time of fertilization to track the precise developmental timing. One day after fertilization (DAF), plants were either kept in control conditions or moved to greenhouse with adjusted HNT. Only the marked developing seeds at 4, 7, and 10 days after fertilization were harvested and analyzed. **(A)** Fresh weight was measured immediately after harvesting. **(B)** For dry weights, the seeds were subjected to drying for 7 days at 40 ˚C prior to measurements. For fresh and dry weight measurements (in mg), sum of ten developing seeds derived from three plants for the respective time-point and treatment was considered. Data is represented as mean ± standard deviation from three independent replicates. Student’s t-test was used for the statistical analysis (***p < 0.001, **p < 0.01, *p < 0.05). HNT, high night-time temperature.

**Figure 2:**
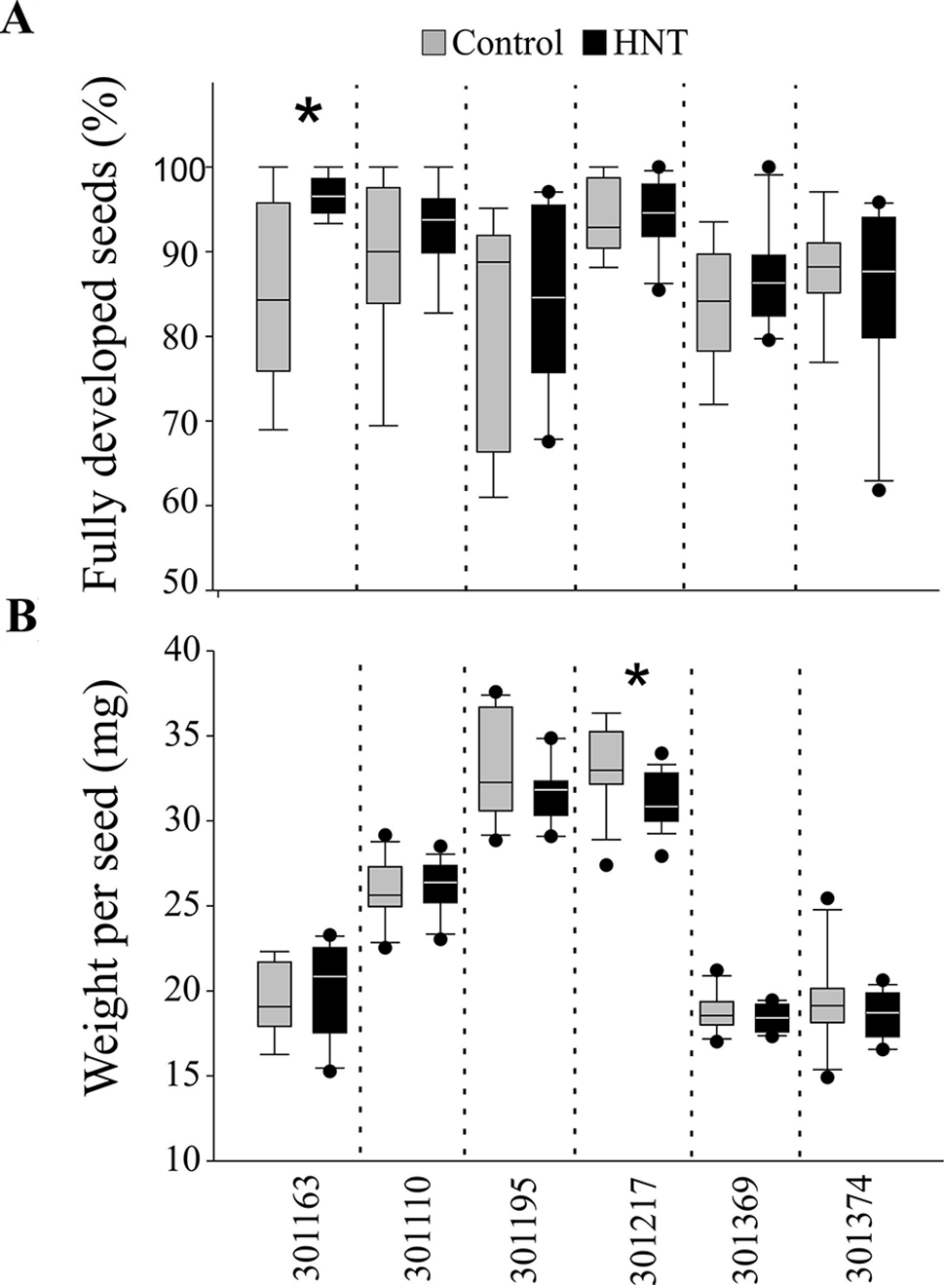
Analysis of mature seeds from control and high night-time temperature treated plants. **(A)** Percentage of fully developed mature seeds. **(B)** Weight per seed (in mg). For this, only the marked florets at the time of fertilization were considered (*n* = 300 – 600) at maturity. One DAF, plants were either kept in control conditions or moved to greenhouse with adjusted HNT until maturity. Data is represented as mean ± standard deviation from eight plants. Student’s t-test was used for statistical analysis (* p < 0.01). HNT: high night-time temperature.

Plants exposed to HNT during seed development showed an overall decrease in fresh weight for two genotypes, 301110 and 301369 (see **Figure 1A**). On the other hand, three genotypes (301195, 301217, and 301374) showed increase in fresh weight for developing seed at 7 and 10 DAF (see **Figure 1A**). For dry weight, three genotypes (301163, 301110, and 301369) showed decrease, while the other three genotypes (301195, 301217, and 301374) showed increase (see **Figure 1B**). These subtle differences during early seed development prompted us to investigate the impact of HNT on mature seeds. For this, we analyzed percentage of fully developed seeds and weight per seed from plants subjected to control and terminal HNT treatment. Except for 301163, we did not observe a significant effect of HNT with respect to number of fully developed seeds (see **Figure 2A**). Moreover, only 301217 showed a significant decrease in weight per seed under HNT compared to the control conditions (see **Figure 2B**). However, morphometric measurements derived from scanning mature seeds indicated that four of the six genotypes showed significant decrease in length/width under HNT conditions (see **Table 2**). These results suggest sensitivity of mature seed morphology to HNT. To explore the effect of HNT on whole plant level, we investigated total number of panicles, total seed number, and total seed weight; none of these parameters were different between control and HNT treated plants (see **Table S1**).

**Table 2:** Morphometric analysis of mature rice seeds.

Genotype	Area (mm^2^)	Perimeter (mm)	Length (mm)	Width (mm)	Length:width
301163 Control 301163 HNT	12.06 ± 1.4 12.33 ± 1.3	15.10 ± 1.2 15.12 ± 1.2	5.81 ± 0.3 5.77 ± 0.3***	2.59 ± 0.2 2.64 ± 0.2	2.26 ± 0.2 2.19 ± 0.1***
301110 Control 301110 HNT	14.50 ± 1.4 14.60 ± 1.3	15.75 ± 0.7 15.74 ± 0.6	5.80 ± 0.2 5.78 ± 0.2	3.14 ± 0.2 3.18 ± 0.2	1.85 ± 0.1 1.83 ± 0.1*
301195 Control 301195 HNT	9.40 ± 2.8 9.82 ± 3.0	12.22 ± 1.9 12.49 ± 2.0	4.39 ± 0.7 4.49 ± 0.7	3.50 ± 0.3 3.51 ± 0.4	1.27 ± 0.2 1.30 ± .02
301217 Control 301217 HNT	14.13 ± 1.4 14.49 ± 1.2*	16.85 ± 1.3 17.14 ± 1.4	6.67 ± 0.5 6.65 ± 0.3	2.73 ± 0.2 2.83 ± 0.2***	2.46 ± 0.3 2.37 ± 0.2*
301369 Control 301369 HNT	12.33 ± 0.9 11.92 ± 0.9***	15.14 ± 0.6 14.87 ± 0.6***	6.04 ± 0.2 5.92 ± 0.2***	2.58 ± 0.1 2.55 ± 0.1***	2.34 ± 0.1 2.32 ± 0.1*
301374 Control 301374 HNT	12.04 ± 1.2 11.91 ± 1.1	16.78 ± 1.1 16.61 ± 1.0*	6.93 ± 0.4 6.88 ± 0.4*	2.15 ± 0.2 2.14 ± 0.2	3.25 ± 0.3 3.24 ± 0.3

Mature seeds from control and HNT treated plants. For statistics, t-test was used *** p < 0.001 and * p < 0.05. Data is represented as mean ± standard deviation from 8 plants, n = 300-600.

### Impacts of HNT on Rice Grain Quality

**Figure 3:**
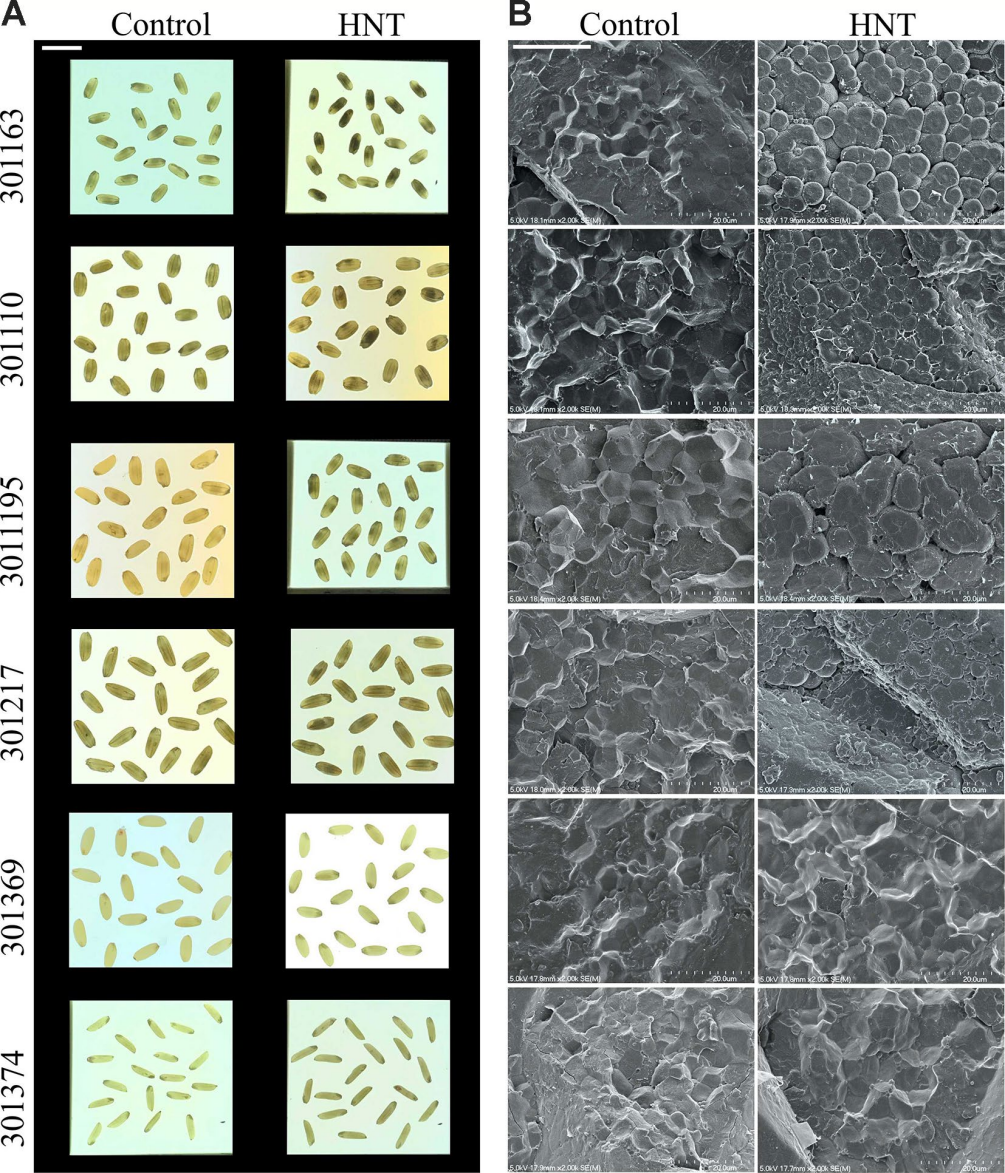
High night-time temperature alters seed quality traits. **(A)** Mature seed images from six rice genotypes subjected to control and HNT conditions (scale: 1 cm). Light box was used to take these images. **(B)** Representative images of scanning electron microscopy (SEM) of mature seed from three independent biological replicates (scale: 20 μm). HNT, high night-time temperature.

We observed visible differences in mature grains between the two temperature treatments; therefore, we examined the seeds with SEM (see **Figure 3A**). Four of the six accessions exhibited abnormal starch granules when grown under HNT treatment during seed development (see **Figure 3B**). The abnormal starch deposition was manifested as loosely packed round shaped starch granules instead of tightly packed polygonal starch granules in seeds from optimal conditions. The loose starch packaging observed in HNT treated plants resulted in numerous pits on the surface of starch granules.

**Figure 4:**
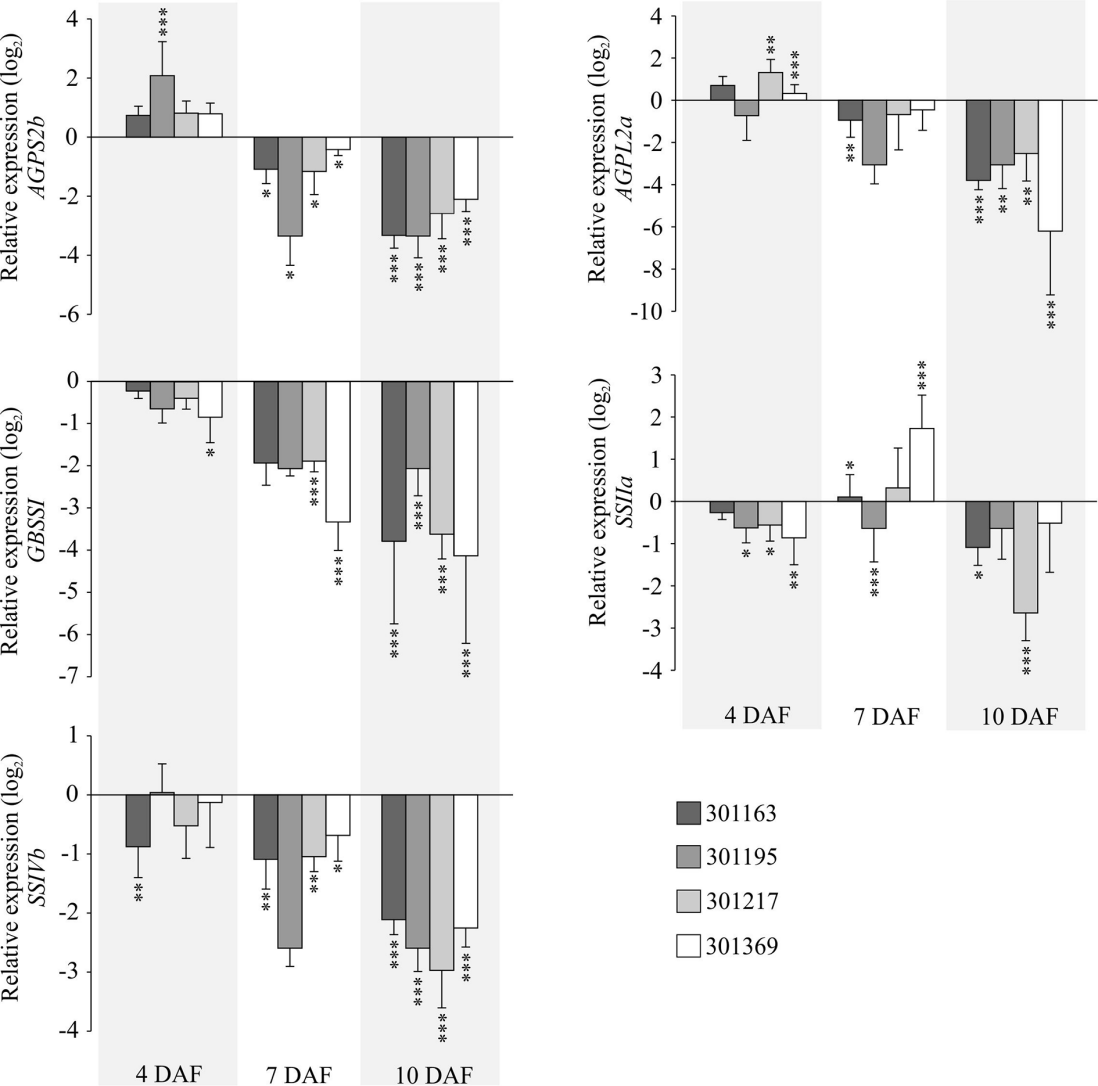
Gene expression analysis of key starch biosynthesis enzymes under HNT. RT-qPCRs representing expression for selected genes related to starch biosynthesis in developing seeds (4, 7, and 10 DAF) corresponding to four genotypes. The relative expression values indicate ratio of HNT to control for the respective genotype and developmental timepoint. For statistics, paired t-test was used to compare expression levels for each gene under HNT relative to control for the respective genotype and developmental timepoint. Error bars indicate standard deviation from three biological and technical replicates. ***indicates *p* < 0.001 and **p < 0.01. DAF, days after fertilization; HNT, high night-time temperature.

To further gain insights into the molecular basis of the observed starch related phenotype, we checked gene expression for a few enzymes involved in starch biosynthesis in developing seed samples (4, 7, and 10 DAF) under HNT for four genotypes (301163, 301195, 301217, and 301369). We analyzed five genes: i and ii) *AGPS2b* and *AGPL2a*, which encode two subunits of ADP-glucose pyrophosphorylase, catalyzes the rate limiting step of starch biosynthesis i.e. the conversion of glucose-1-phosphate and ATP to ADP-glucose and pyrophosphate, iii) granule bound starch synthase (GBSSI), also known as *Waxy*, which is involved in amylose biosynthesis, and iv and v) two starch synthase genes (*SSIIa* and *SSIVb*) involved in amylopectin biosynthesis. Except *SSIIa*, all genes were upregulated in the tested genotypes at 7 and 10 DAF relative to 4 DAF under control conditions; however, the magnitude of upregulation varied for individual gene and genotype (see **Figure S3**). Under HNT conditions, all genes (except *AGPS2b* and *AGPL2a* at 4 DAF and *SSIIa* at 7 DAF) were downregulated in the tested genotypes and developmental stages (see **Figure 4**). The HNT induced downregulation of these genes possibly impairs starch biosynthesis leading to starch structural abnormalities in the mature seeds (see **Figure 3B**).

### Metabolite Profiles in Developing Rice Seeds

**Figure 5:**
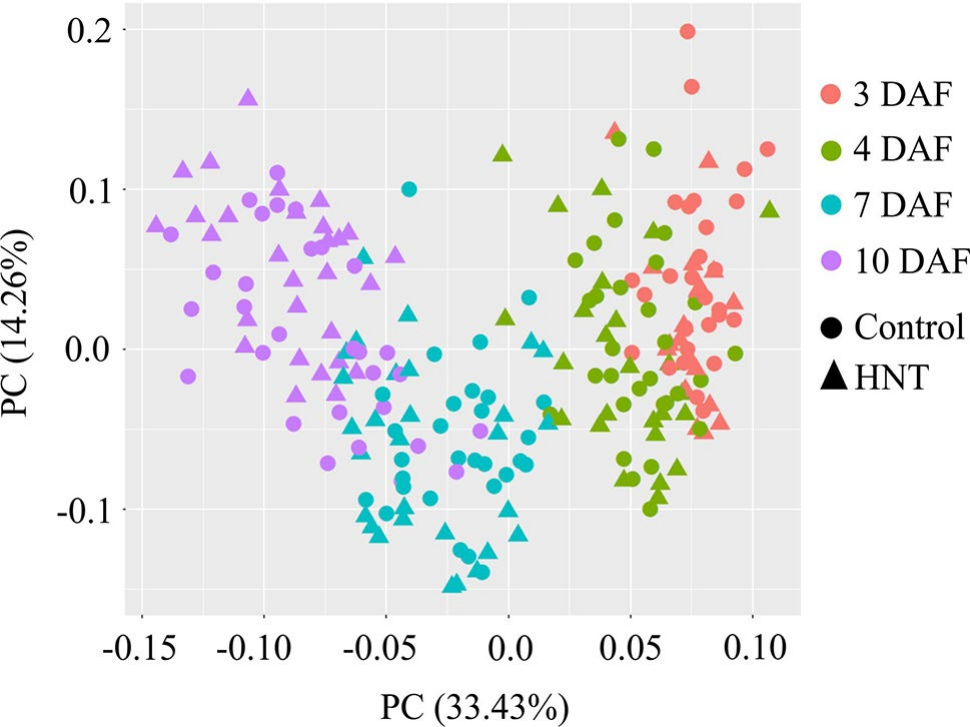
Principal component analysis (PCA) of the metabolite profiles of rice seed development under control and high night-time temperature conditions. Orange, green, blue, and violet colors represent 3, 4, 7, and 10 days after fertilization (DAF). Circle and triangle represent metabolome for control samples and high night temperature treated samples. Five independent biological replicates were used for the metabolomic analysis. Each point corresponds to one replicate from each of the six rice genotypes. HNT, high night-time temperature.

Metabolic profiling of the developing rice seeds using GC-MS detected 57 metabolites. The detected are metabolites comprised of 23 sugars and derivatives, 16 organic acids, 16 amino acids, 2 secondary metabolites, and others (1). Principal component analysis (PCA) was used to determine the dynamic metabolite patterns in developing rice seeds under control and HNT (see **Figure 5**). In total, 47.8% of variation among all samples was explained by principal components 1 and 2 (PC1: 33.4%, PC2: 14.2%). Distinct metabolite patterns for early (3 and 4 DAF) and late (7 and 10 DAF) time-points were detected. The metabolite profiles from 3 DAF were more closely related to 4 DAF, suggesting little differences in their metabolic contents; this is likely due to the short time interval (1 day) between the two respective stages. For the 7 and 10 DAF time points, which coincide with accumulation of starch reserves (Yamagata et al., 1982), a clear separation in metabolite profile was detected (see **Figure 5**).

### Dynamics of Metabolite Profile During Early Seed Development in Rice

**Figure 6:**
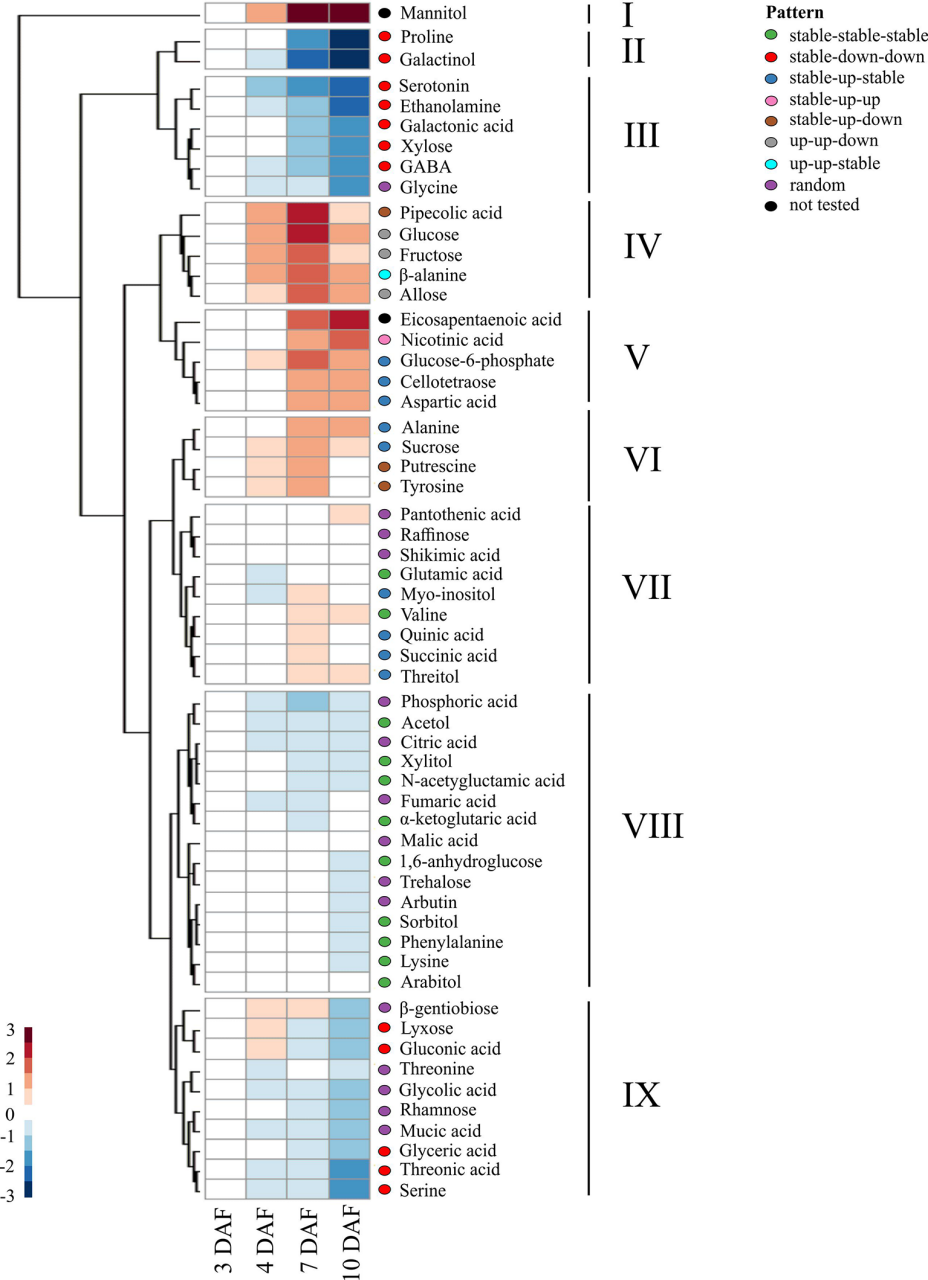
Temporal trends in the levels of metabolites across early seed development. A heatmap showing the changes in levels of metabolites during the early seed development in the control condition. The ratio of the median of normalized peak area at each time point against the initial time point (3 DAF) was log2 transformed to calculate relative metabolite levels. Hierarchical clustering analysis was performed using Euclidean distance to group metabolites with similar temporal patterns. Directional patterns represent statistically significant changes in metabolite level relative to metabolite level at previous timepoint using generalized linear models, using a cutoff in adjusted p-value at 0.05 (**Table S3**), which are represented by colored ellipses to the right of the heatmap.

We first examined the metabolites from developing seed under control conditions to determine the extent of metabolic changes during early seed development in rice. For this, metabolite data from the six rice accessions were pooled to find developmental patterns that were widely conserved across sub-species. Hierarchical cluster analysis (HCA) of log2 transformed median ratios of metabolite levels at 4, 7, and 10 DAF to those at 3 DAF was performed. The results were visualized using a heatmap to find groups of metabolites that shared patterns of accumulation across early grain development (see **Figure 6**). Additionally, linear models were used with log transformed data to determine statistically significant patterns of directional changes (see **Table S2**).

The nine clusters identified using HCA could largely be explained in terms of the directional patterns determined using generalized linear models over the developmental time-period (see **Figure 6**). Here, we described the temporal change as either “up”, “down”, or “stable” when a metabolite increased, decreased, or did not show changes in contents in comparison to the previous time point, respectively. Cluster I was populated by mannitol, which showed a consistent accumulation across time; however, its pattern was not statistically characterized because inconsistent accumulation for the respective metabolite was detected among different genotypes. The metabolites in clusters II, III, and IX showed a decline with the progression of seed development (3 to 10 DAF). These clusters contained proline, galactinol, serotonin, ethanolamine, galactonic acid, xylose, γ-aminobutyric acid (GABA), lyxose, gluconic acid, glyceric acid, thronic acid, and serine (**Table S2**). Clusters IV and VI represented a pattern where metabolites continued to accumulate beyond 3 DAF, peaked around 7 DAF, and then started to decline until at least 10 DAF with directional patterns of up-up-down, stable-up-down, up-up-stable, and stable-up-stable. These clusters contained pipecolic acid, glucose, fructose, β-alanine, allose, sucrose, putrescine, and tyrosine. Cluster V showed a consistent increase in content across time and was represented by stable-up-stable and stable-up-up directional patterns corresponding to 4, 7, and 10 DAF, respectively. This cluster contained nicotinic acid, glucose-6-phosphate, cellotetraose, and aspartic acid. Clusters VII and VIII represented metabolites whose contents were mostly stable through the developmental time-points. This group contained shikimic acid, quinic acid, and certain intermediates from the citric acid cycle: citric acid, fumaric acid, α-ketoglutaric acid, and malic acid. A sugar trehalose was clustered within this group.

### Impact of HNT on Metabolites During Early Seed Development

**Figure 7:**
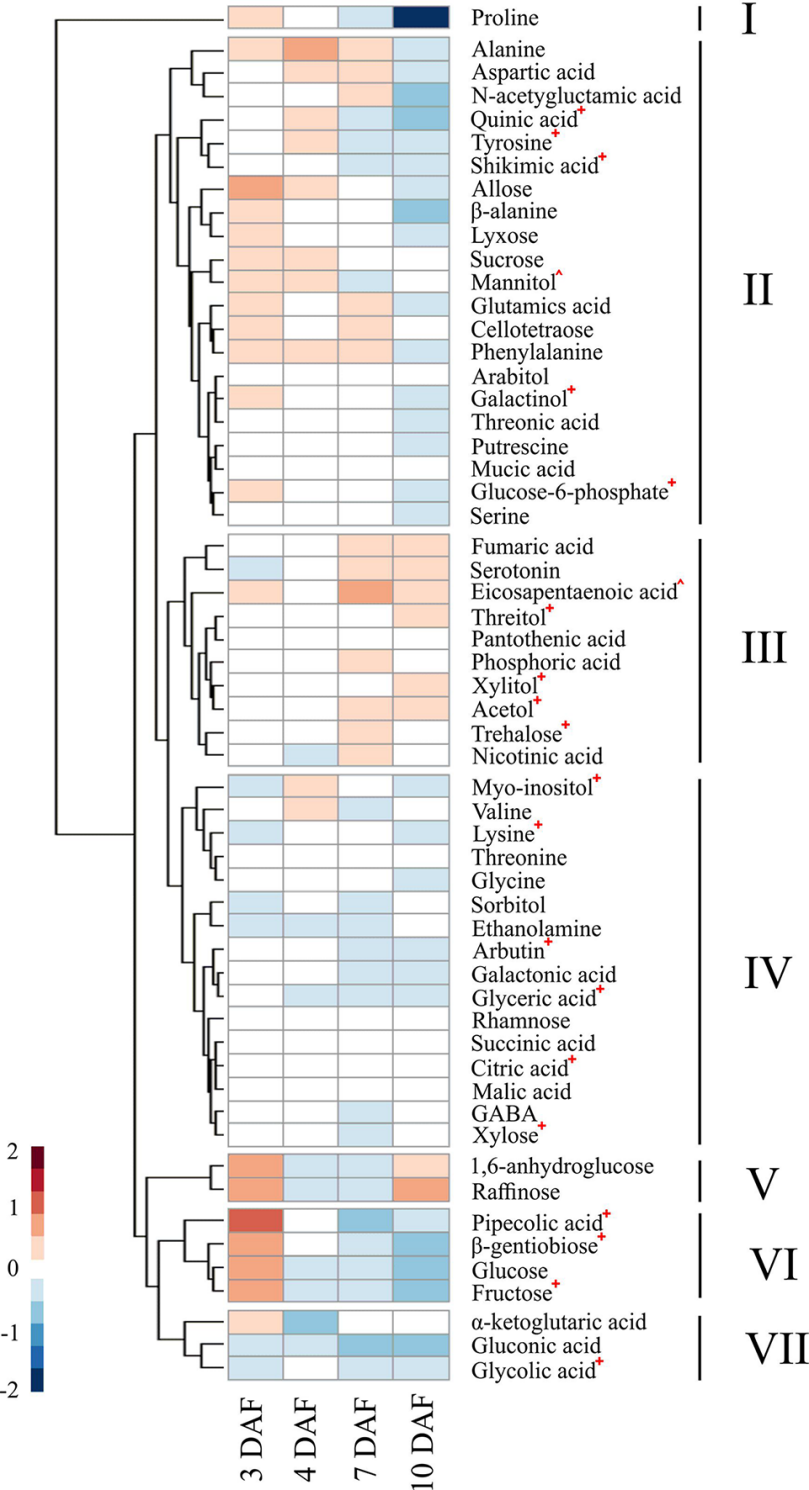
Effects of high night-time temperature on the levels of metabolites across early seed development. A heatmap showing the effects of HNT on the levels of individual metabolites during the early seed development. The ratio of the median of normalized peak area under HNT against control conditions at each time point was log_2_ transformed to calculate relative metabolite levels. Hierarchical clustering analysis was performed using Euclidean distance to group metabolites similarly affected by HNT treatment. Statistically significant difference in metabolite levels between HNT and control conditions was determined by meta-analysis, using a cutoff in adjusted p-value at 0.05 (**Table S4**) and was denoted by a +, ^ indicates a metabolite which was not statistically tested due to inconsistency among genotypes. HNT, high night-time temperature.

We explored how warmer nights affect the dynamic metabolic patterns during early seed development. Metabolite profiles of developing seeds were analyzed at 3, 4, 7, and 10 DAF from plants grown under control and HNT conditions during grain development. All the tested genotypes exhibited consistency with respect to the metabolic profiles in response to HNT, as no genotype-specific alterations were detected. Data from six accessions were pooled for elucidating the shared metabolic responses of developing rice seeds. Median of metabolite levels under HNT condition were divided by the values from control samples for a respective time point to calculate the ratio of metabolite levels between HNT and control groups. This median ratio was then log2 transformed, so that the ratio of relative metabolite levels in the plants under HNT against control conditions could be seen at each time point. HCA of these median ratios revealed 7 clusters of metabolites showing differential patterns in dynamic responses across early seed development (see **Figure 7**; **Table S3**). The effects of HNT on the metabolite trajectories were assessed by meta-analysis of the genotype-specific *limma* models (**Table S3**). Here, only the metabolites in each cluster that showed statistically significant differences in the trajectory are discussed. Five of the seven clusters contained metabolites that showed statistically significant HNT effects. Proline was the only member in cluster I and its levels decreased as seed development progressed. Cluster II represented a large cluster where metabolite levels were higher under HNT than control conditions in early time points (3 and 4 DAF), with the ratio decreasing at later time points (7 and 10 DAF). This group was comprised of metabolites associated with the shikimic acid pathway; quinic acid, tyrosine, and shikimic acid. It also included glucose-6-phosphate and galactinol. Metabolites in cluster III represented a pattern where the levels of metabolites in HNT and control plants remained the same from 3 DAF to 4 DAF; then, from 7 DAF to 10 DAF, metabolite levels under HNT condition increased disproportionately to control. This cluster included the sugar alcohols: threitol, xylitol, acetol, as well as the sugar trehalose. Clusters IV and VII were characterized by metabolites whose levels under HNT conditions were lower than controls across all four time points. These clusters included myo-inositol, lysine, arbutin, glyceric acid, citric acid, xylose, and glycolic acid. Cluster VI represented a pattern where metabolite levels under HNT conditions were elevated relative to control conditions at 3 DAF and the pattern reversed at 10 DAF. This cluster included pipecolic acid, β-gentiobiose, and fructose.

### RT-qPCR Analysis of Genes Associated With Amino Acid, Sugar, and Shikimate Metabolism

**Figure 8:**
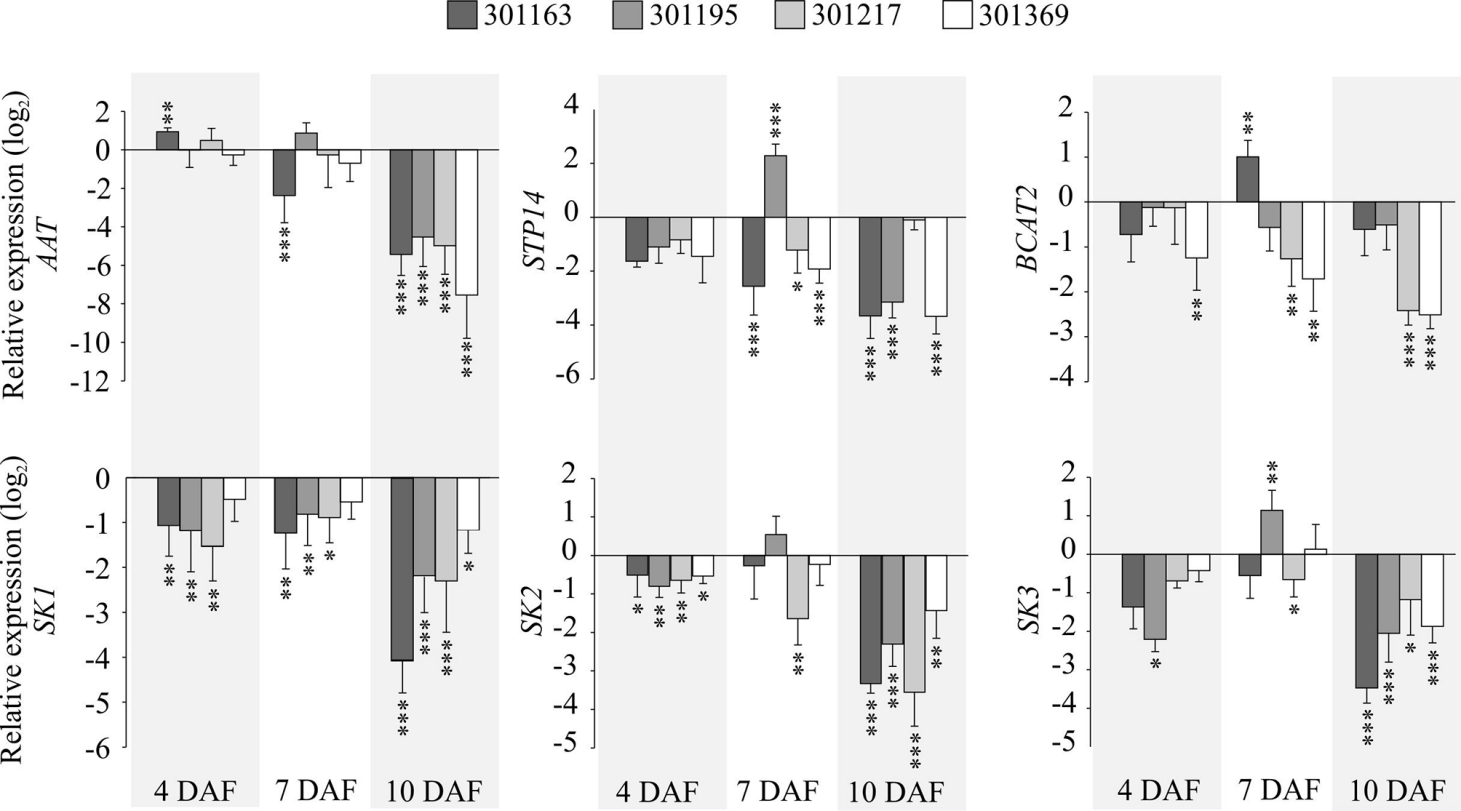
Gene expression analysis of genes associated with selected metabolites under HNT. RT-qPCRs representing expression for selected genes: aspartate aminotransferase (*AAT),* sugar transporter (*STP14*), branched chain amino acid transaminase 2 (*BCAT2*), and shikimate kinases (*SK1*, *SK2*, and *SK3*) in developing seeds (4, 7, and 10 DAF) corresponding to four genotypes. The relative expression values indicate ratio of HNT to control for the respective genotype and developmental timepoint. For statistics, paired t-test was used to compare expression levels for each gene under HNT relative to control for the respective genotype and developmental timepoint. Error bars indicate standard deviation from three biological and technical replicates. ***indicates *p* < 0.001, **p < 0.01 and *p < 0.05. DAF, days after fertilization; HNT, high night-time temperature.

We checked the expression for selected genes associated with metabolic pathways of amino acid, sugar, and shikimate in the developing seeds. We examined i) Aspartate Amino Transferase (*AAT*) that catalyzes formation of aspartate from oxaloacetate, ii) a sugar transporter, *STP14* transports monosaccharide sugars (glucose, galactose, and mannose) and exhibits strong expression in endosperm (Poschet et al., 2010), (iii) a branched chain amino acid transferase, *BCAT2* that is involved in metabolism of valine, leucine, and isoleucine (Diebold et al., 2002), and (iv) shikimate kinases (*SK1*, *SK2,* and *SK3*) that are known to catalyze phosphorylation of shikimate to shikimate-3-phosphate in the shikimate pathway and are preferentially expressed in panicles (Kasai et al., 2005). *AAT, STP14*, and *BCAT2* showed upregulation at 7 and 10 DAF relative to 4 DAF under control conditions (see **Figure 4**). These three genes exhibited decreasing trend in their expression from 4 to 10 DAF under HNT relative to control (see **Figure 8**). On the other hand, all three shikimate kinases are upregulated at 7 DAF and downregulated at 10 DAF relative to 4 DAF under control conditions (see **Figure 4**); under HNT, we detected downregulation of shikimate kinases in developing seeds (4, 7, and 10 DAF) relative to their respective controls (see **Figure 8**). The magnitude of downregulation increased with the progression of seed development (10 DAF > 7 DAF > 4 DAF; see **Figure 8**).

## Discussion

### HNT Alters Mature Seed Size and Quality

The morphological consequence of HNT on rice in the post-zygotic, reproductive stage is the change in seed size, especially the length/width ratio of mature seeds (see **Table 2**). This could be a consequence of change in the timing of endosperm developmental events, which are known to be highly sensitive to temperature. Even a transient high temperature stress during early seed development perturbs the developmental timing (Folsom et al., 2014; Chen et al., 2016). Specifically, transition of endosperm from the syncytial to cellularization is a key event in terms of initiation of starch and protein accumulation as well as other metabolites. For example, moderate (35°C/30°C; day and night) and severe heat stress (39°C/34°C; day and night) causes precocious and delayed endosperm cellularization, respectively (Folsom et al., 2014; Chen et al., 2016). Despite prominent differences in seed length/width, only one genotype showed distinction with respect to weight per seed under HNT (see **Figure 2B**). This is possibly because the HNT treatment initiated during early seed development, which primarily defines seed length (Folsom et al., 2014; Chen et al., 2016), while seed width is determined in relatively later developmental stages; grain filling. On the other hand, weight of seed is not only the function of length/width but also depends on density of the packed storage compounds. Thus, decrease in the length to width ratio does not necessarily reflect alteration in seed weight. No other differences were observed between control and HNT treatment with respect to end-point measurements at the whole plant level (see **Table S1**). These findings are in contrast with other reports concluding that rice yield-related parameters are compromised during HNT (Morita et al., 2005; Cheng et al., 2009; Mohammed and Tarpley, 2011; Shi et al., 2013; Coast et al., 2015). This could be because we analyzed only the precisely marked seeds at the time of fertilization for examining the phenotypic and metabolic consequences of a relatively mild increase in night-time temperature (+5°C). We did not include florets that fertilized during the HNT treatment. This ensured that we were focusing on the impact of HNT on post-zygotic seed development and not on heat sensitivity of pollination or the fertilization event.

Chalkiness is an undesirable feature of rice seed as it impairs the overall appearance of milled rice and makes the rice prone to breakage during the milling process (Lisle et al., 2000; Kadan et al., 2008). Chalky grains are characterized by appearance of opaque spots in the endosperm (Fitzgerald et al., 2009). We observed the chalkiness phenotype in seeds from HNT treated plants, which prompted us to analyze mature seed using SEM. We detected abnormality in the packaging of starch granules under HNT relative to control (see **Figure 3**), which is in line with previous reports linking high temperature stress to chalky grain formation. Further, gene expression analysis for key starch biosynthesis genes revealed their significant downregulation by 10 DAF under HNT (see **Figure 4**). Previous work also discusses reduction in activity of key starch biosynthesis enzymes under high temperature stress (Sato and Inaba, 1976; Cheng et al., 2005; Yamakawa et al., 2007; Ishimaru et al., 2009). Thus, our observation of abnormal starch granules is associated with HNT induced downregulation of the key starch biosynthesis enzymes. In summary, we conclude that despite the minor effect of HNT on overall rice yield parameters, we observed that HNT negatively impact seed quality. Further experiments focusing on starch quantification in developing and mature seeds can widen our understanding of HNT impact on grain quality parameters.

### Groups of Metabolites Show Temporal Trends Across Early Seed Development

Hu et al., 2016 described the dynamics of 214 metabolites, comprised of amino acids, dipeptides, lipids, and flavonoids, from four diverse rice cultivars at 7, 10, 14, 28, and 42 DAF. Except flavonoids, they observed a universal drop in metabolite levels between 7 and 10 DAF. This was explained by the decline in metabolite concentrations caused by the transition of endosperm from cell growth, division, and morphogenesis phases to grain filling phase when metabolites are consumed to synthesize storage carbohydrates and proteins, which is inferred from transcriptomic and proteomic data (Xu et al., 2008; Sato et al., 2011; Deng et al., 2013; Hu et al., 2016). In addition to the early grain-filling stage (7 and 10 DAF), our study included developmentally critical time-points (3 and 4 DAF) that correspond to the process of endosperm cellularization. Our dataset suggests that a single decreasing pattern from 7 to 10 DAF reported by Hu et al., 2016, could be partitioned into two distinct profiles: i) 3 to 4 DAF and ii) 4 to 7 DAF (see **Figure 5**). In the first pattern, grouped as clusters IV and VI, metabolite levels increased from 3 to 7 DAF and then declined at 10 DAF, exhibiting up-up-down, stable-up-down, or up-up-stable directional trends. Cluster VI contains sucrose, which accumulated until day 7, similar to previous findings by (Singh and Juliano, 1977). Cluster IV contains the two major breakdown products of sucrose—glucose and fructose (hexoses). This observation is in line with the fact that sucrose is transported from photosynthetic leaf tissue (source) to the developing seed (sink). The incoming sucrose undergoes catabolic reactions, thereby resulting in increased hexoses content. The increased content of hexoses serves as the major carbon sources to the metabolic pathways including glycolysis, oxidative pentose phosphate pathway, and the tricarboxylic acid cycle to produce building blocks of cellular macromolecules (Gibson, 2005; Jeon et al., 2010), as well as for starch biosynthesis in the grain filling stage. Moreover, the accumulated glucose is required to provide energy to sustain rapid mitotic divisions during early seed development (Sinclair and de Wit, 1975). In this context, we detected an increased transcript abundance for monosaccharide sugar transporter (*STP14*) with the progression of seed development (10 DAF > 7 DAF > 4 DAF; see **Figure S3**), which is probably linked to the increased sugar demand. Furthermore, consistent with previous studies, we detected that a non-reducing sugar, raffinose, starts accumulating by 10 DAF in rice (see **Figure 7**). Previous studies have reported the role of raffinose during later stages of seed development in preventing embryo desiccation as it progresses towards physiological maturity (Downie et al., 2003; Sengupta et al., 2015). Another non-reducing sugar trehalose, which is involved in embryo development (Eastmond et al., 2002), was not altered during the examined developmental stages. A second pattern in clusters II, III, and IX represented a continuous decrease in the concentration of metabolites already underway at 4 DAF and continuing through 7 and 10 DAF with directional patterns of stable-down-stable, stable-down-down, or stable-stable-down. This group was comprised of the amino acids, proline, glycine, serine, and threonine, all of which showed changes consistent with Hu et al., 2016 (see **Figure 7**). Other amino acids, including β-alanine, aspartic acid, alanine, glutamic acid, valine, lysine, N-acetylglutamic acid, and phenylalanine, showed stable or decreasing trend after 7 DAF, although the decline was not always statistically significant (see **Figure 7**). Protein contents and total free amino acids are known to increase till around 8 DAF per grain basis (Cruz et al., 1970). Nevertheless, our results suggested that the contents of amino acids decreased during early seed development per fresh weight basis. One possible explanation for this contradiction is a dilution of amino acids during the grain expansion phase. As the volume of developing seeds increases disproportionately to amino acid levels, amino acid concentration may decrease, even as the count of amino acids per grain increases. This inability of amino acid influx to keep up with grain expansion may cause a deficiency that limits cell division, which is active in this developmental stage and determines grain sink capacity.

### HNT Induces Metabolic Perturbation Across Early Seed Development

We observed elevated hexose levels under HNT conditions at 3 DAF and then decreased relative to the control at 10 DAF. Although this change in accumulation under HNT was similar for glucose and fructose, it was statistically significant only for fructose. The decrease in hexoses at 10 DAF coincides with significant reduction in dry weight of developing seeds by 10 DAF (see **Figure 1B**); however, this decrease in dry weight might not be enough to explain the final yield parameters At the transcript level, *STP14* showed downregulation at 10 DAF under HNT (see **Figure 8**), which corroborates with the levels of the detected monosaccharides (see **Figure 7**). Unlike hexoses, levels of trehalose differed significantly between HNT and control groups (HNT > control; see **Figure 7**). Trehalose levels often reflect the status of sucrose metabolism and trehalose-6-phosphate. The latter is a reaction intermediate in the conversion pathway between sucrose and trehalose and serves as a signaling molecule involved in carbon partitioning and plant development (Ponnu et al., 2011). Similarly, sugar alcohols accumulated to a greater extent under HNT during early stages of grain filling (7 and 10 DAF). These results are comparable to changes observed in wheat spikes where accumulation of sugars and their alcohol derivatives were observed under HNT (Impa et al., 2019). Amino acids like alanine and phenylalanine also showed increased abundance till 7 DAF under HNT. This is in line with wheat spike metabolite results from Impa et al., 2019 where increased accumulation of alanine was observed under HNT conditions. These similarities further suggest conserved metabolic response in cereals under HNT conditions.

**Figure 9:**
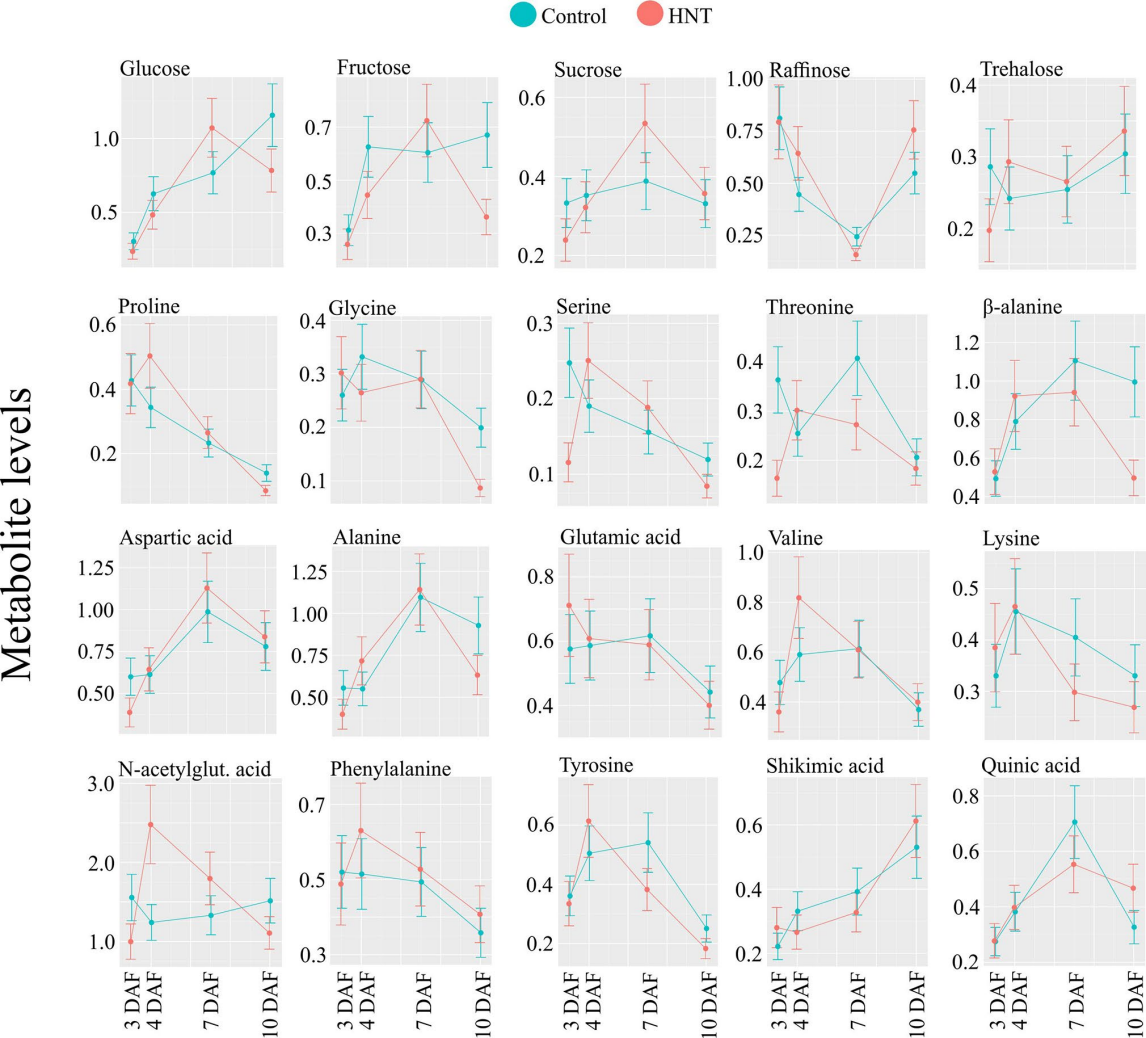
Abundance levels for selected metabolites. Mean of the abundance levels from six rice genotypes at the respective seed developmental stage under control and HNT (other metabolites are shown in **Figure S2**). Data is represented as mean ± standard deviation from five biological replicate for each genotype and treatment. HNT, high night-time temperature.

Metabolites related to the shikimic acid pathway including tyrosine, shikimic acid and quinic acid, were also affected significantly by the HNT treatment (see **Figure 9**). These metabolites grouped together within cluster II in HCA shown as **Figure 7**. The three metabolites also showed similar patterns in temporal accumulation during early seed development **Figure 5**. These results suggest that activity of the shikimic acid pathway alters during grain development and is affected by HNT. Gene expression analysis revealed downregulation of shikimate kinases by 7 and 10 DAF under HNT (see **Figure 8**). This observation correlates with declining levels for metabolites associated with the shikimic acid pathway (quinic acid, tyrosine, and shikimic acid) by 7 and 10 DAF under HNT (**Figure 7** and **Figure S4**). Previous literature has shown that the shikimic acid pathway contribute to biotic and abiotic stress response through the synthesis of protective compounds such as anthocyanins and tocopherols (Dewick, 1995). It also produces the precursors for a phytohormone, salicylic acid, which is involved in the transduction of response to several abiotic stresses such as high and low temperatures as well as heavy metal stress (Janda et al., 2007). Although levels of phenylalanine were not significantly affected by HNT, it has been suggested that tyrosine can feed into the phenylpropanoid pathway *via* Tyr ammonia-lyase (Neish, 1961). The shikimic acid pathway is also involved in synthesis of auxin, which is related to plant development and response to abiotic stresses including high UV light tolerance (He and Li, 2001). Double knock-out mutants of shikimate kinase genes (*ASK1* and *ASK2*) in *Arabidopsis* show delayed embryogenesis due to defective cell division and cell expansion (Liu et al., 2004). Also, the shikimate kinase genes in rice (*OsSK1*, *OsSK2,* and *OsSK3*) show preferential expression at the panicle heading stage that makes this pathway an interesting one to explore for its role during seed development (Kasai et al., 2005) and HNT response in rice.

Mis-regulation of the TCA cycle have been reported in response to HNT in leaves (Glaubitz et al., 2017); however, we did not find significant differences in metabolites associated with the TCA cycle in developing seed indicating that the effects of HNT on metabolism differ between tissues. We did detected accumulation of aspartate in response to HNT, which is similar to the response of leaf tissue under HNT (Glaubitz et al., 2017). Aspartate accumulation is probably related with suppression of *AAT* expression under HNT since this enzyme catalyzes reversible transamination between glutamate and oxaloacetate to generate aspartate and 2-oxoglutarate (de la Torre et al., 2014). Increased activity of AAT related genes has been previously reported under HNT and this enhancement has been linked to more nitrogen metabolism and absorption in the leaves and stem under HNT (Kanno et al., 2009 and Zhou et al., 2009). Certain amino acids that originate from aspartate (asparagine, methionine, and threonine) have shown increased accumulation under HNT conditions in leaf tissue (Glaubitz et al., 2017), but we did not detect any change in other aspartate family amino acids in our study. These results indicate that the effects of HNT in aspartate metabolism likely have tissue specificity.

## Conclusion

In summary, our study characterizes the sensitivity of mature seed morphology and quality under HNT; however, contrary to the previous literature we observed subtle differences in final yield parameters. These contrasting results are probably because of different methodology and temperature treatments used to evaluate the phenotypic response. In our study, we focused on the effect of HNT on post-zygotic seed development and not on pollination or fertilization. This approach helped us to avoid over-estimating the impact of HNT on yield related parameters. Although, yield related parameters were not impacted severely, the metabolic effect of HNT during rice seed development was clear. The commonality between metabolic profile of wheat and rice in response to HNT with effects on grain yield highlights the importance of sugar metabolism during early grain development. Moreover, the significant increase in metabolites with respect to HNT offers a strong case for characterizing their roles under high temperature stress by gene editing studies. In this study, we evaluated a limited number of genotypes so we cannot generalize the HNT response in rice; thus, future experiments involving a larger set of diverse rice genotypes will lead to a better understanding of molecular and physiological responses to HNT in rice that can potentially be used to develop thermotolerant rice varieties.

## Data Availability Statement 

All datasets generated for this study are included in the article/**Supplementary Material**.

## Author Contributions 

PP and HW conceived and designed the experiment. PP, BD, NA, and JS performed the experiments. KH and QZ performed statistical analysis. PP, BD, NA, and TO wrote the manuscript. All authors read and approved the manuscript.

## Funding

This work was supported by National Science Foundation Award # 1736192 to HW and TO.

## Supplementary Material

Supplementary Figure 1 The high night-time temperature regime. Plants were grown under control conditions (16 h light/8 h dark at 28 ± 1 ˚C and 23 ± 1 ˚C) till flowering. Florets were marked at the time of fertilization and transferred to either control or high night temperature (HNT; 16 h light/8 h dark at 28°C/28°C) conditions. For metabolite profiling analysis, developing seed tissue (with husk) was collected from 3, 4, 7 and 10 DAF from control and HNT treated plants. Fresh and dry weight of the developing seed tissue from 4, 7, and 10 DAF was also determined from control and HNT treated plants. End-point measurements (percentage of fully developed seeds, weight per seed, total number of panicles, total number of seeds per plant, and total seed weight per plant) were performed at physiological maturity of the rice plant. Terminal HNT and control treatments were given to the respective plants. HNT: high night-time temperature.

Supplementary Figure 2 Abundance levels for metabolites. Mean of the abundance levels from six rice genotypes at the respective seed developmental stage under control and HNT. Data is represented as mean ± standard deviation from five biological replicate for each genotype and treatment. HNT: high night-time temperature.

Supplementary Figure 3 Gene expression analysis of key starch biosynthesis enzymes under control. RT-qPCRs representing expression for selected genes related to starch biosynthesis. The analysis was performed on developing seeds (4, 7, and 10 DAF) corresponding to four genotypes under control. The values at 4 DAF were used as baseline to calculate relative expression for 7 and 10 DAF for the respective genotype. For statistics, paired t-test was used to compare expression levels for each gene at 7 and 10 DAF relative to 4 DAF under control for the respective genotype. Error bars indicate standard deviation from three biological and technical replicates. *** indicates *p*< 0.001 and ** *p*< 0.01. DAF: days after fertilization. HNT: high night-time temperature.

Supplementary Figure 4 Gene expression analysis of genes associated with selected metabolites under control. RT-qPCRs representing expression for selected genes: aspartate aminotransferase (*AAT),* sugar transporter (*STP14*), branched chain amino acid transaminase 2 (*BCAT2*), and shikimate kinases (*SK1*, *SK2*, and *SK3*) on developing seeds (4, 7, and 10 DAF) corresponding to four genotypes under control. The values at 4 DAF were used as baseline to calculate relative expression for 7 and 10 DAF for the respective genotype. For statistics, paired t-test was used to compare expression levels for each gene at 7 and 10 DAF relative to 4 DAF under control for the respective genotype. Error bars indicate standard deviation from three biological and technical replicates. *** indicates *p*< 0.001 and ** *p*< 0.01. DAF: days after fertilization. HNT: high night-time temperature.
