# Supplementary figures and images for: Metabolic Dynamics of Developing Rice Seeds Under High Night-Time Temperature Stress
Source: Front Plant Sci. 2019 Nov 8;10:1443. doi: 10.3389/fpls.2019.01443 (PMC6857699; doi:10.3389/fpls.2019.01443)

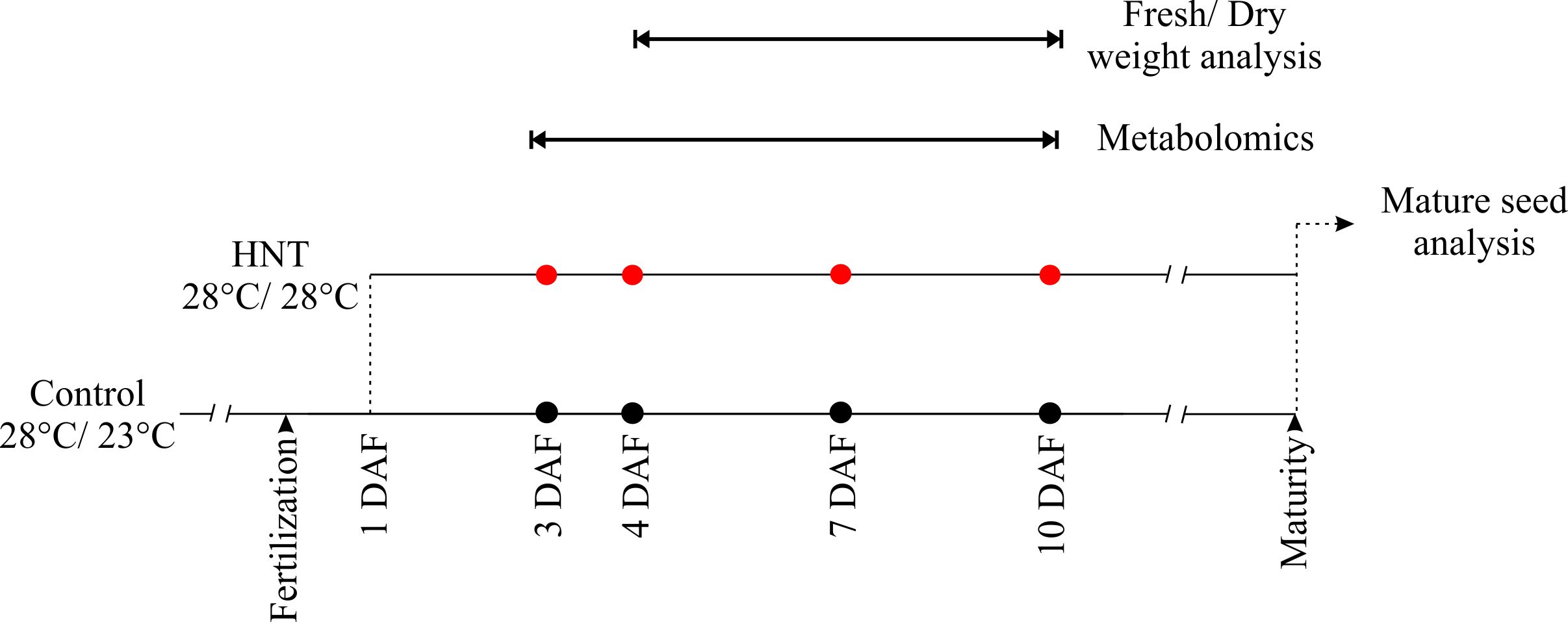

Supplement: Supplementary Figure 1 — The high night-time temperature regime. Plants were grown under control conditions (16 h light/8 h dark at 28 ± 1 ˚C and 23 ± 1 ˚C) till flowering. Florets were marked at the time of fertilization and transferred to either control or high night temperature (HNT; 16 h light/8 h dark at 28°C/28°C) conditions. For metabolite profiling analysis, developing seed tissue (with husk) was collected from 3, 4, 7 and 10 DAF from control and HNT treated plants. Fresh and dry weight of the developing seed tissue from 4, 7, and 10 DAF was also determined from control and HNT treated plants. End-point measurements (percentage of fully developed seeds, weight per seed, total number of panicles, total number of seeds per plant, and total seed weight per plant) were performed at physiological maturity of the rice plant. Terminal HNT and control treatments were given to the respective plants. HNT: high night-time temperature. [file Image_1.jpeg]

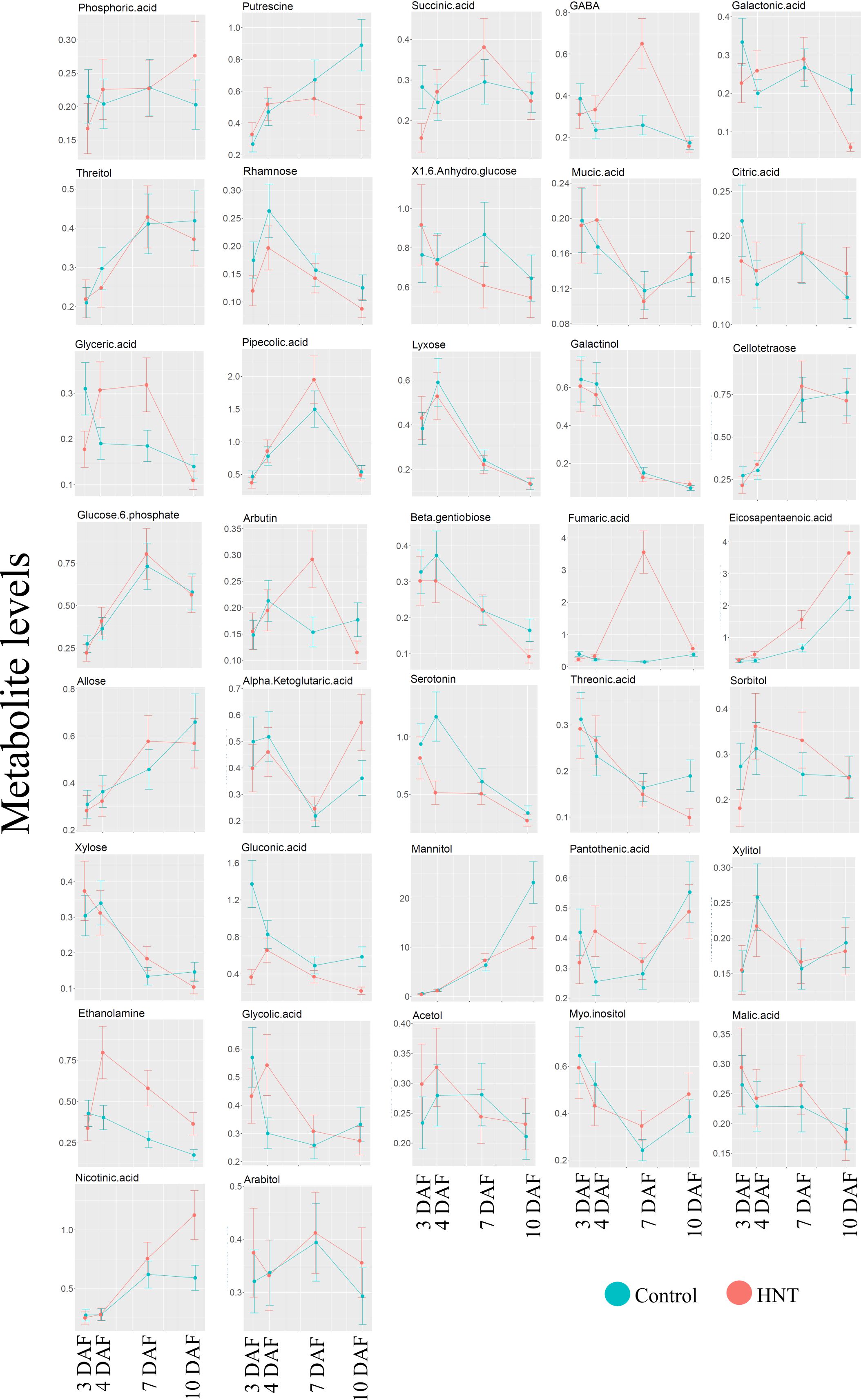

Supplement: Supplementary Figure 2 — Abundance levels for metabolites. Mean of the abundance levels from six rice genotypes at the respective seed developmental stage under control and HNT. Data is represented as mean ± standard deviation from five biological replicate for each genotype and treatment. HNT: high night-time temperature. [file Image_2.jpeg]

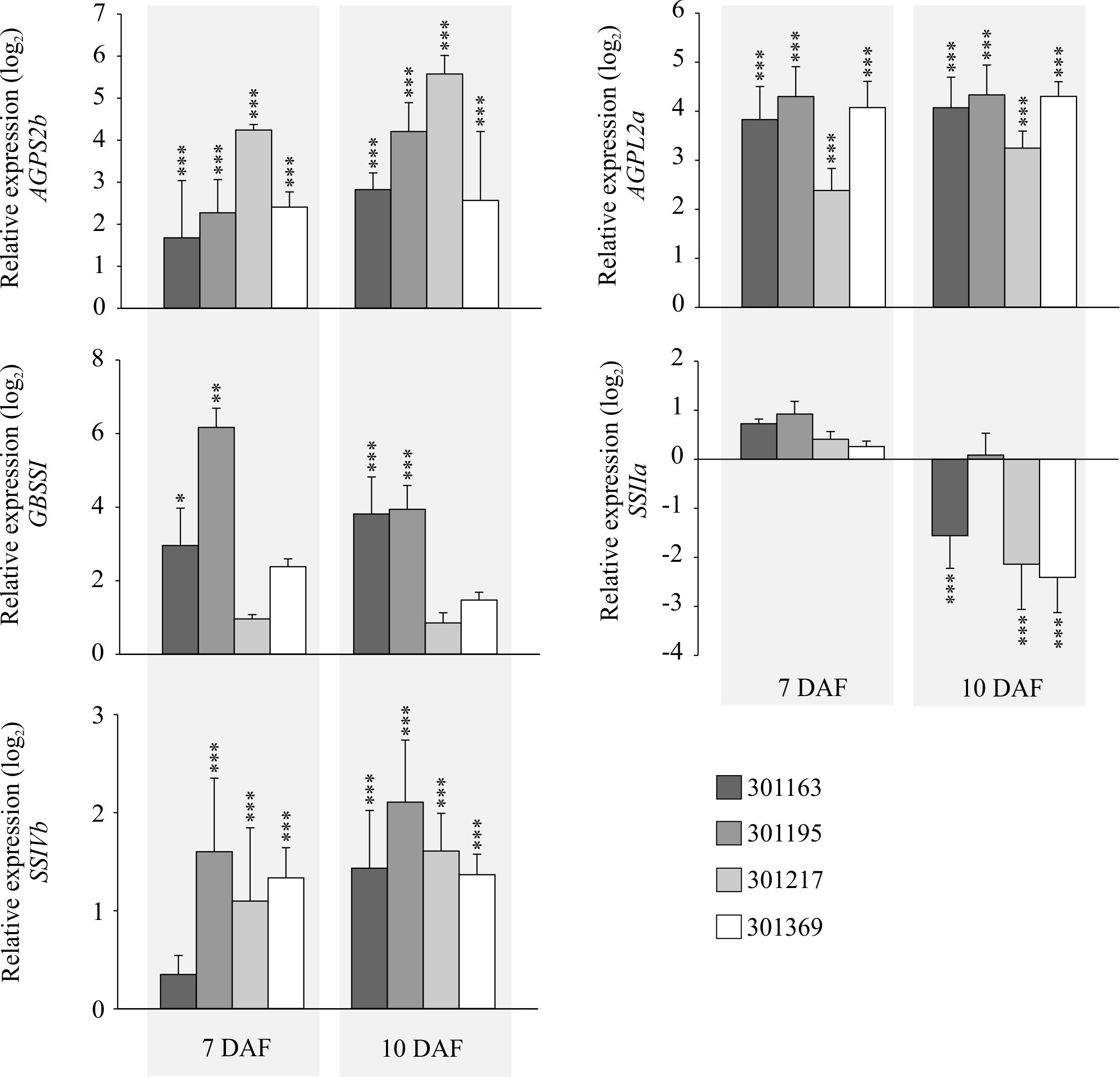

Supplement: Supplementary Figure 3 — Gene expression analysis of key starch biosynthesis enzymes under control. RT-qPCRs representing expression for selected genes related to starch biosynthesis. The analysis was performed on developing seeds (4, 7, and 10 DAF) corresponding to four genotypes under control. The values at 4 DAF were used as baseline to calculate relative expression for 7 and 10 DAF for the respective genotype. For statistics, paired t-test was used to compare expression levels for each gene at 7 and 10 DAF relative to 4 DAF under control for the respective genotype. Error bars indicate standard deviation from three biological and technical replicates. *** indicates p< 0.001 and ** p< 0.01. DAF: days after fertilization. HNT: high night-time temperature. [file Image_3.jpeg]

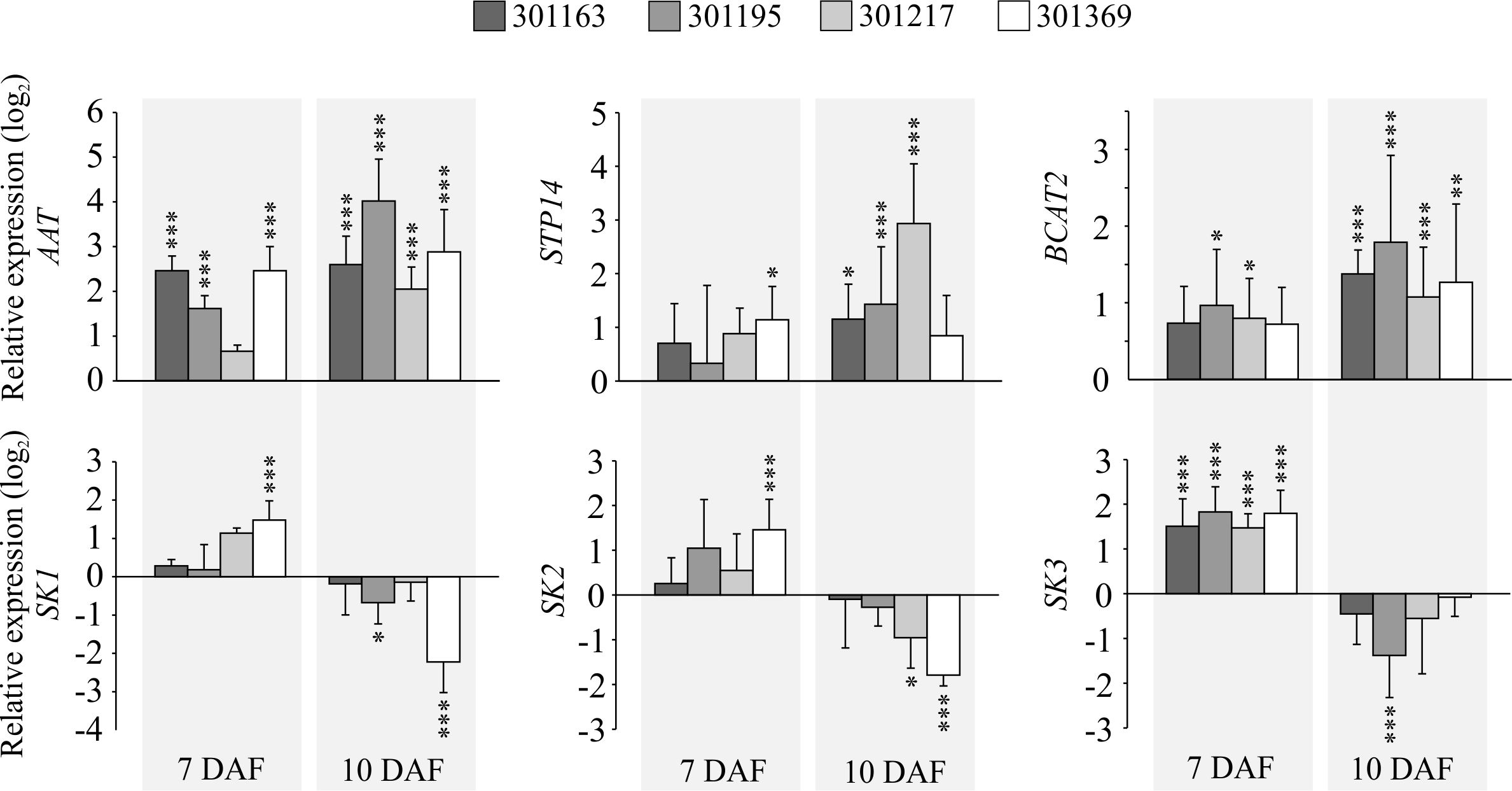

Supplement: Supplementary Figure 4 — Gene expression analysis of genes associated with selected metabolites under control. RT-qPCRs representing expression for selected genes: aspartate aminotransferase (AAT), sugar transporter (STP14), branched chain amino acid transaminase 2 (BCAT2), and shikimate kinases (SK1, SK2, and SK3) on developing seeds (4, 7, and 10 DAF) corresponding to four genotypes under control. The values at 4 DAF were used as baseline to calculate relative expression for 7 and 10 DAF for the respective genotype. For statistics, paired t-test was used to compare expression levels for each gene at 7 and 10 DAF relative to 4 DAF under control for the respective genotype. Error bars indicate standard deviation from three biological and technical replicates. *** indicates p< 0.001 and ** p< 0.01. DAF: days after fertilization. HNT: high night-time temperature. [file Image_4.jpeg]
